# Supplementary material for: Getting More Out of Biomedical Documents with GATE's Full Lifecycle Open Source Text Analytics
Source: PLoS Comput Biol. 2013 Feb 7;9(2):e1002854. doi: 10.1371/journal.pcbi.1002854 (PMC3567135; doi:10.1371/journal.pcbi.1002854)
Supplement: Dataset S1 — GATE software. Dataset S1 bundled with this paper contains a distribution of GATE (or see http://gate.ac.uk/download/). (TGZ) [file pcbi.1002854.s001.tgz › plos-gate/plugins/Gazetteer_LKB/doc/general.html]

The Large KB gazetteer - Questions and Answers


---

Last Published: 2009-10-17

---

##### Large KB Gazetteer

- Overview
- Usage
- **Q & A**
- GATE Compatibility
- GATE Gazetteer Feature Comparison
- Semantic Enrichment PR
- Samples

##### Developer Documentation

- Dictionary Lifecycle

## Questions and Answers

**General**

1. Why is a file called kim.trusted.entities.cache created in the dictionary folder?
2. Can I extract data from any RDF database?
3. Can I extract data from a relational database?

**Installation**

1. How do I install the Large KB Gazetteer?
2. Can I install the gazetteer in GATE 5.0 ?

## General

Why is a file called kim.trusted.entities.cache created in the dictionary folder?
:   kim.trusted.entities.cache contains a binary data structure that contains the dictionary
    extracted according to the dictionary settings. The name of the file is kept for
    backwards compatibility with the Ontotext KIM Plaftorm. Note that the file
    snapshot.properties is a plain text file which contains metadata that, along with
    the configuration, describe the contents of the binary dictionary.

    ---

Can I extract data from any RDF database?
:   The Large KB gazetteer communicates with RDF databases through the standard open-source Sesame 2 API.
    The database connection is configured through *config.ttl* in the dictionary directory which contains standard Sesame
    configuration template. Note that in order to
    configure a new database, you will need to add to the classpath the database Sesame adapter.

    Here are some databases that should work correctly with the gazetteer.

    - Mulgara (formerly known as Kowari) - through the
      OpenRDF Mulgara SAIL
    - OpenLink Virtuoso - through the
      Virtuoso Sesame Adapter
    - Franz Inc. AllegroGraph - through the Sesame 2 SAIL adapter supplied
      with the AllegroGraph distribution.
    - Jena - through RDF2Go

    ---

Can I extract data from a relational database?
:   You should convert your relational data into simple RDF. Try Triplify.

## Installation

How do I install the Large KB Gazetteer?
:   The gazetteer and its companion processing resources form a standard CREOLE plugin, compatible with GATE 5.1. See the  CREOLE plugin usage guide in the GATE documentation.

    ---

Can I install the gazetteer in GATE 5.0 ?
:   The gazetteer requires GATE 5.1, but a GATE 5.0 installation can be modified so it becomes compatible. (That is how we tested the gazetteer before the 5.1 release). See the GATE compatibility guide for more information.

---

©
2009
Ontotext AD

---
